# Herbal Cocktail Hydrogel Film (*Garcinia mangostana*, *Centella asiatica*, and *Chromolaena odorata* Extracts): A Novel Wound Dressing Approach

**DOI:** 10.1155/ijbm/5576769

**Published:** 2026-01-31

**Authors:** Tanikan Sangnim, Chonlada Panpipat, Suwisit Manmuan, Nontanat Leehueng, Wasutthanat Suphan, Chanapa Thuenaram, Kampanart Huanbutta

**Affiliations:** ^1^ Faculty of Pharmaceutical Sciences, Burapha University, Chonburi, 20131, Thailand, buu.ac.th; ^2^ Piboonbumpen Demonstration School, Burapha University, Chonburi, 20131, Thailand, buu.ac.th; ^3^ Department of Manufacturing Pharmacy, College of Pharmacy, Rangsit University, Pathum Thani, 12000, Thailand, rsu.ac.th

**Keywords:** anti-inflammatory, antibacterial activity, herbal extract hydrogel, hydrogel film, wound healing

## Abstract

Chronic and infected wounds remain a significant clinical challenge due to delayed healing and the risk of microbial contamination. Conventional wound dressings often fail to provide comprehensive therapeutic support, necessitating the development of advanced multifunctional materials. This study aimed to develop a multifunctional hydrogel wound dressing incorporating herbal extracts from *Garcinia mangostana*, *Centella asiatica*, and *Chromolaena odorata*, targeting key biological functions essential for wound healing. The novelty lies in the synergistic combination of these three extracts to address multistage healing needs and the systematic optimization of the polymer matrix via a design‐of‐experiments (DOE) approach. The extracts were obtained through optimized extraction techniques and quantified using HPLC, confirming the presence of bioactive markers. Pharmacological evaluations revealed distinct and synergistic activities: *Garcinia mangostana* extract showed potent antibacterial effects against *Staphylococcus aureus* and *Staphylococcus epidermidis* (MIC of 7.8 μg/mL) and strong antioxidant capacity (ABTS IC_50_ of 26.5 μg/mL). In contrast, *Centella asiatica* extract demonstrated high biocompatibility on human keratinocytes (IC_50_ of 736.11 μg/mL), while *Chromolaena odorata* provided significant anti‐inflammatory and hemostatic benefits. A hydrogel base was developed using a polyvinyl alcohol (PVA)/sodium carboxymethyl cellulose (SCMC) polymer matrix, optimized via factorial design for texture, swelling ratio, and moisture retention. The extract‐loaded hydrogel maintained skin‐compatible pH, showed improved tensile strength and flexibility, and exhibited superior swelling capacity (188.7%) compared to the blank (120.3%). Overall, the formulated hydrogel demonstrates promise as an effective wound dressing with antibacterial, anti‐inflammatory, and antioxidant properties, suitable for chronic or infected wound applications.

## 1. Introduction

A wound can be described as a defect or a break in the skin, resulting from physical or thermal damage or as a result of the presence of an underlying medical or physiological condition. Wounds, whether acute or chronic, pose a significant global healthcare burden due to delayed healing and increased risk of infection. Acute wounds are tissue injuries that typically heal completely, with minimal scarring, within the expected 8‐ to 12‐week timeframe [1]. Chronic wounds, conversely, are tissue injuries that heal slowly, meaning they have not healed beyond 12 weeks [2]. A 2018 retrospective analysis of Medicare beneficiaries identified that about 8.2 million people had wounds with or without infections [3]. The selection of appropriate wound dressing plays a pivotal role in promoting tissue regeneration and preventing microbial contamination. However, conventional dressings such as gauze, foams, and bandages often fail to maintain a moist environment, provide sustained drug release, or conform to irregular wound surfaces [4]. These limitations have prompted the advancement of modern wound care systems, incorporating active pharmaceutical ingredients or herbal extracts into wound dressings, or employing novel dressing formats that foster a favorable healing environment, enhance biocompatibility, and facilitate the targeted delivery of therapeutic agents directly to the wound site [5, 6].

As shown in Table 1, there has been growing interest in integrating herbal medicines into modern wound care systems due to their diverse and potent pharmacological properties, including anti‐inflammatory, antimicrobial, antioxidant, and wound healing activities [7]. The incorporation of herbal extracts into wound dressings offers several advantages over synthetic drugs, such as lower toxicity, reduced risk of adverse effects, improved biocompatibility, and the presence of multiple bioactive compounds that can act synergistically to promote tissue regeneration and accelerate the healing process. Herbal extracts are often preferred over synthetic drugs in wound dressings due to their natural origin, multitarget therapeutic actions, and reduced likelihood of inducing antimicrobial resistance [8]. Moreover, an emerging trend in advanced wound therapy is the use of combination herbal extracts, which may provide enhanced therapeutic efficacy through synergistic interactions among their constituents, offering a broader spectrum of activity and improved healing outcomes [9]. In this context, the present study selected three medicinal plants, *Garcinia mangostana* (mangosteen peel), *Centella asiatica* (gotu kola), and *Chromolaena odorata* (siam weed), based on their well‐documented efficacy in supporting tissue regeneration and infection control in wound healing. *Garcinia mangostana* is rich in xanthones, particularly α‐mangostin, which exhibits potent antimicrobial, anti‐inflammatory, and antioxidant activities [10] that help reduce bacterial burden and oxidative stress at the wound site [11]. *Centella asiatica* contains triterpenoids such as asiaticoside and madecassoside, which are known to stimulate fibroblast proliferation [12], collagen synthesis [13], and angiogenesis [13], thereby promoting granulation tissue formation and re‐epithelialization. *Chromolaena odorata* is abundant in flavonoids like apigenin and quercetin, contributing to its hemostatic [5], antimicrobial [14], and cell‐proliferative properties [15] that enhance wound contraction and tissue repair. The synergistic pharmacological actions of these plant‐derived compounds offer a multifaceted approach to wound management by accelerating healing, minimizing infection, and improving tissue regeneration, making them promising candidates for incorporation into advanced herbal‐based wound dressing systems.

**TABLE 1:** Comparative summary of related herbal‐based hydrogel or wound dressing studies.

Study	Extracts/active agents	Polymer base/formulation	Key bioactivities	Distinctive findings/limitations
Phumlek et al. [16]	Mangosteen (*Garcinia mangostana*)	Carrageenan, locust bean gum/patch	Antibacterial, anti‐inflammatory	Mainly for acne, not wound dressing; tested single extract
Vamvanij et al. [17]	*Aloe vera*, *Centella asiatica*, *Allium cepa*	Chitosan base/hydrogel	Antioxidant, antimicrobial, epithelium regeneration	Focused on combination, but limited in various bioactivities for wound healing
Huanbutta et al. [7]	Propolis extract	PVA/film‐forming system	Antimicrobial, antioxidant	Single extract; limited mechanical optimization
Sangnim et al. [5]	*Chromolaena odorata*	Plastoid® B, liquid plaster	Anti‐inflammatory, hemostatic	No mechanical optimization of the film system or multiextract combination
Bylka et al. [12]	*Centella asiatica*	Ointment, cream, and gel	Promoted cellular proliferation and collagen synthesis	Single‐function activity; lacks antimicrobial function
Panawes et al. [18]	*Garcinia mangostana*	Alginate‐coated gauze wound dressing	Antimicrobial and antioxidant	Mangosteen extract enhanced antibacterial activity with controlled release, but only limited concentrations were tested and no synergistic combinations explored.

To serve as a suitable carrier for the herbal extracts, hydrogel films were selected due to their advantageous physicochemical and biological properties ideal for wound care. Hydrogels are three‐dimensional, crosslinked hydrophilic polymer networks that absorb significant volumes of wound exudate while maintaining integrity, providing a moist environment that facilitates tissue regeneration and accelerates healing [19]. Their transparency allows for visual inspection of the wound without disturbing the dressing [20], and their high‐water content supports the controlled release of incorporated bioactive agents [21]. Additionally, hydrogels are known for their biocompatibility [22], nonadherence to the wound [23], and capacity to minimize trauma upon removal. In this study, a composite hydrogel matrix was formulated using polyvinyl alcohol (PVA) for its film‐forming and mechanical properties, and sodium carboxymethyl cellulose (SCMC) for its bioadhesiveness and water retention capabilities [24]. Glycerin was added as a plasticizer to improve flexibility and comfort during application, particularly on irregular skin surfaces. This hydrogel system was optimized to deliver sustained release of the herbal extracts while maintaining optimal mechanical strength, hydration, and conformability, thereby creating an effective and patient‐friendly wound dressing platform.

Although numerous studies have incorporated individual herbal extracts into hydrogels for wound healing, few have explored a tri‐herbal system designed through statistical optimization. The present study is the first to integrate *Garcinia mangostana*, *Centella asiatica*, and *Chromolaena odorata* in a single hydrogel platform. Therefore, this research aims to contribute to the advancement of next‐generation wound dressings by integrating medicinal plant‐based therapeutics with modern biomaterial technologies. Firstly, the pharmacological properties of each selected herbal extract (*Garcinia mangostana*, *Centella asiatica*, and *Chromolaena odorata*) were thoroughly examined, focusing on their antibacterial, anti‐inflammatory, and antioxidant activities, which are critical for effective wound healing. Following the characterization of these bioactivities, hydrogel film formulations were systematically developed using a design‐of‐experiments (DOE) approach to optimize key parameters such as polymer composition, plasticizer content, and herbal extract loading. This strategy enabled the identification of optimal formulation conditions that ensure desirable mechanical strength, swelling capacity, and controlled release of active compounds. The resulting hydrogel films were then evaluated for their physicochemical properties, drug release behavior, and biological efficacy, with the ultimate goal of creating a safe, effective, and patient‐friendly herbal‐based wound dressing system suitable for clinical application.

## 2. Materials and Methods

### 2.1. Materials

Glycerin (≥ 99.5% purity) was purchased from Global Green Chemical Co., Ltd. (Thailand). PVA was obtained from Carlo Erba Reagents S.r.l. (Italy), and SCMC was procured from Krungthepchemi Co., Ltd. (Thailand). Dried pericarps of *Garcinia mangostana* were sourced from Trat Province, Thailand, while *Centella asiatica* and *Chromolaena odorata* were harvested from Chonburi Province, Thailand. All plant materials were collected between November and December 2024 and authenticated prior to use.

### 2.2. Herbal Extract Preparation

#### 2.2.1. *Garcinia mangostana*

The extraction of compounds from mangosteen rind was performed using the Soxhlet extraction method with 95% ethanol as the solvent. The mangosteen rinds were thoroughly washed, air‐dried, and further dried in a hot air oven at 40°C until completely dry. The dried rinds were then ground into a fine powder. A total of 40 g of the powdered rind was weighed and placed into a porous thimble, which was then inserted into the Soxhlet extraction tube. Subsequently, 400 mL of 95% ethanol (1 : 10 w/v ratio) was added to the extraction apparatus. The solvent was allowed to cycle through the porous thimble containing the plant powder and collect in a round‐bottom flask. The extraction was carried out at 75°C for 15 h. After completion, the extract was allowed to cool and was filtered using Whatman No. 1 filter paper. The filtrate was concentrated by evaporating the solvent using a rotary vacuum evaporator at 40°C, followed by freeze‐drying. The final extract was weighed, and the extraction yield was calculated as the percentage of extract weight relative to the dry plant material.

#### 2.2.2. *Centella asiatica*

*Centella asiatica* leaves were washed thoroughly and dried in a hot air oven at 40°C until completely dry. The dried leaves were then ground into a fine powder. A sample of 25 g of the powdered leaves was weighed and mixed with 1000 mL of 95% ethanol (1 : 40 w/v ratio). The mixture was extracted using an ultrasonicator for 1 h at 60°C. The extract was filtered through Whatman No. 1 filter paper. The resulting filtrate was concentrated by evaporating the solvent using a rotary vacuum evaporator at 40°C. The concentrate was further dried using a freeze dryer. The weight of the final extract was recorded, and the extraction yield was calculated as a percentage of the extract weight relative to the dry plant weight.

#### 2.2.3. *Chromolaena odorata*

*Chromolaena odorata* leaves were thoroughly washed and dried in a hot air oven at 40°C. The dried leaves were then ground into a fine powder. A sample of 200 g of the powdered leaves was weighed and mixed with 2000 mL of 95% ethanol (1 : 10 w/v ratio). The mixture was macerated for 24 h. After maceration, the extract was filtered using Whatman No. 1 filter paper. The resulting filtrate was concentrated by evaporating the solvent using a rotary vacuum evaporator at 40°C, and the concentrate was subsequently freeze‐dried. The weight of the final extract was recorded, and the extraction yield was calculated as the percentage of extract weight relative to the dry plant material.

### 2.3. Evaluation of Herbal Extract

#### 2.3.1. Phytochemical Analysis

##### 2.3.1.1. *Garcinia mangostana*

α‐Mangostin was selected as a bioactive marker. A mangosteen pericarp extract solution was prepared at a concentration of 1 mg/mL using methanol as the solvent. All solutions were filtered through a 0.45‐μm membrane filter and transferred into 1‐mL vials for HPLC analysis. The analysis of α‐mangostin in the extract was performed using a Shimadzu HPLC system equipped with an LC‐20AD pump, an autosampler, and an SPD‐20A UV detector. Separation was carried out on a Phenomenex LC C18 column (4.6 × 250 mm, 5 μm), maintained at 25°C. The mobile phase consisted of a gradient elution using acetonitrile and 0.1% ortho‐phosphoric acid at a flow rate of 1.0 mL/min. The injection volume was 20 μL, and UV detection was performed at 320 nm. The total runtime for each injection was 37 min. The gradient elution profile was as follows: 70% acetonitrile and 30% 0.1% ortho‐phosphoric acid from 0.00 to 15.00 min; increased to 75% acetonitrile and 25% acid at 18.00 min; followed by 80% acetonitrile and 20% acid from 19.00 to 25.00 min; and returned to the initial ratio of 70:30 from 26.00 to 37.00 min.

##### 2.3.1.2. *Centella asiatica*

Madecassoside, asiaticoside, madecassic acid, and asiatic acid were selected as marker compounds for the *Centella asiatica* extract. The analysis was performed using the HPLC technique based on the method established in our previous study [25]. The HPLC system consisted of a Shimadzu instrument equipped with an LC‐20AD pump, an autosampler, and an SPD‐20A UV detector. Chromatographic separation was achieved on a Phenomenex LC C18 column (4.6 × 250 mm, 5 μm), maintained at 25°C. The mobile phase consisted of a gradient elution using acetonitrile and 0.3% ortho‐phosphoric acid at a flow rate of 1.0 mL/min. The injection volume was 20 μL, and UV detection was conducted at a wavelength of 205 nm. The total runtime for each injection was 32 min. The gradient elution program was as follows: 30% acetonitrile and 70% 0.3% ortho‐phosphoric acid from 0.00 to 3.00 min; increased to 70% acetonitrile and 30% acid from 11.00 to 25.00 min; and returned to the initial ratio from 27.00 to 32.00 min.

##### 2.3.1.3. *Chromolaena odorata*

Gallic acid, quercetin, and apigenin were selected as biomarkers for *Chromolaena odorata* extract. The standards were prepared at concentrations ranging from 0.006 to 0.1 mg/mL. A solution of *Chromolaena odorata* leaf extract was prepared at a concentration of 1 mg/mL using methanol as the solvent. All solutions were filtered through a 0.45‐μm membrane filter and transferred into 1‐mL vials for HPLC analysis. The analysis of active constituents in the extract was performed using a Shimadzu HPLC system equipped with an LC‐20AD pump, an autosampler, and an SPD‐20A UV detector. Chromatographic separation was carried out using a Phenomenex LC C18 column (4.6 × 250 mm, 5 μm) maintained at 25°C. The mobile phase consisted of an isocratic elution using a solvent mixture of methanol, acetonitrile, acetic acid, phosphoric acid, and water in a volumetric ratio of 200 : 100 : 10 : 10 : 200. The flow rate was set at 0.6 mL/min, and the injection volume was 10 μL. UV detection was performed at 272 nm, and the total runtime for each injection was 30 min.

#### 2.3.2. Antioxidant Activity

The antioxidant activity of all herbal extracts was assessed using three established in vitro assays: the ABTS (2,2′‐azinobis‐[3‐ethylbenzthiazoline‐6‐sulfonic acid]) radical cation decolorization assay, the DPPH (1,1‐diphenyl‐2‐picrylhydrazyl) free radical scavenging assay, and the ferric reducing antioxidant power (FRAP) assay. These assays were employed to evaluate the extracts’ capacity to donate electrons or hydrogen atoms for the neutralization of free radicals and to determine their overall reducing power, thus providing a comprehensive characterization of antioxidant potential. The detailed procedures for each assay are described in the following sections.

##### 2.3.2.1. 2,2′‐Azinobis‐(3‐ethylbenzothiazoline‐6‐sulfonic Acid) (ABTS) Assay

The antioxidant activity of the extracts was evaluated using the ABTS radical cation decolorization assay. A 7 mM ABTS stock solution was prepared and allowed to stand in the dark at room temperature for 18 h to generate the ABTS•^+^ radical. Serial dilutions of each extract (4–64 μg/mL) were prepared, and 100 μL of each concentration was added in triplicate to a 96‐well microplate. Subsequently, 100 μL of the ABTS radical solution was added to each well. The plate was incubated in the dark at room temperature for 6 min, after which the absorbance was measured at 734 nm using a microplate reader. Ascorbic acid served as the positive control. The percentage of radical scavenging activity was calculated using the following equation:

(1) % inhibition=AbsABTS−AbssampleAbsABTS−Abscontrol×100.

##### 2.3.2.2. 2,2‐Diphenyl‐1‐picrylhydrazyl (DPPH) Free Radical Scavenging Assay

The antioxidant capacity of the extracts was assessed using the DPPH free radical scavenging assay. A 0.2 mM DPPH solution was freshly prepared in methanol. Various concentrations of each extract (20–320 μg/mL) were prepared, and 100 μL of each concentration was added in triplicate to a 96‐well microplate. An equal volume (100 μL) of the DPPH solution was then added to each well. The plate was incubated in the dark at room temperature for 30 min. After incubation, absorbance was measured at 517 nm using a microplate reader. Ascorbic acid was used as the reference standard. The percentage of radical scavenging activity was calculated according to equation (1).

##### 2.3.2.3. FRAP Assay

The FRAP of the herbal extracts was determined using the FRAP assay. The FRAP reagent was freshly prepared by mixing 300 mM acetate buffer (pH 3.6), 20 mM ferric chloride (FeCl_3_) solution, and 10 mM 2,4,6‐tripyridyl‐s‐triazine (TPTZ) solution in a 10 : 1 : 1 (v/v/v) ratio and protected from light. Extract solutions were prepared at concentrations ranging from 20 to 320 μg/mL. For each concentration, 50 μL of extract was added in triplicate to a 96‐well microplate, followed by 100 μL of FRAP reagent. The mixture was incubated at room temperature for 10 min. Deionized water was used as the blank. Absorbance was measured at 593 nm using a microplate reader. Trolox was used as the standard reference compound. Antioxidant activity was expressed as percentage inhibition and calculated using equation (1). The results were further represented as IC_50_ values, indicating the concentration of the extract required to inhibit 50% of the oxidative activity.

#### 2.3.3. Antibacterial Activity

##### 2.3.3.1. Minimal Inhibitory Concentration (MIC)

The MIC representing antimicrobial activity of the extracts was evaluated using the broth dilution method. The test microorganisms used in this study included two Gram‐positive bacterial strains: *Staphylococcus aureus* (Methicillin‐Resistant Staphylococcus aureus [MRSA]) and *Staphylococcus epidermidis*. Bacterial inocula were prepared by transferring 2–3 colonies grown on trypticase soy agar (TSA) plates into liquid culture media and incubating at 37°C for 16–18 h. The bacterial suspensions were then adjusted to a turbidity equivalent to the 0.5 McFarland standard (approximately 10^8^ CFU/mL) using 0.85% NaCl solution. The adjusted inoculum was subsequently diluted 1:99 in broth medium to achieve the working concentration. Extracts of *Centella asiatica* were prepared in final concentrations ranging from 0.125 to 4 mg/mL, while extracts of mangosteen pericarp and *Chromolaena odorata* leaves were prepared at final concentrations ranging from 0.0039 to 1 mg/mL, using dimethyl sulfoxide (DMSO) as the solvent. Then, MIC was determined by adding 1.98 mL of inoculated culture medium and 0.02 mL of the extract solution into the same test tube. The mixture was thoroughly mixed and incubated at 37°C for 16–18 h. Amoxicillin at a concentration of 0.03 mg/mL was used as the positive control, while 0.85% NaCl solution served as the negative control.

##### 2.3.3.2. Minimum Bactericidal Concentrations (MBC)

The test tube containing the lowest concentration from the MIC assay that showed no visible bacterial growth was streaked onto solid agar medium. The plate was then incubated at 37°C for 24 h. The MBC was determined by observing the absence of bacterial growth on the agar surface. The MBC value was recorded in micrograms per milliliter (μg/mL). All experiments were conducted in five replicates to ensure reproducibility and statistical reliability.

#### 2.3.4. Anti‐Inflammatory Activity

The anti‐inflammatory activity of the extracts was evaluated by measuring nitric oxide (NO) inhibition in lipopolysaccharide (LPS)‐stimulated RAW 264.7 macrophage cells. NO production was quantified using the modified Griess method to assess the extracts’ efficacy in reducing inflammation responses [26].

RAW 264.7 murine macrophage cells were purchased from the American Type Culture Collection (ATCC; Catalog No. TIB‐71) and cultured in RPMI‐1640 medium supplemented with 10% fetal bovine serum (FBS), penicillin (100 units/mL), and streptomycin (100 μg/mL) at 37°C in a 5% CO_2_ incubator. All cell lines were routinely tested and confirmed to be free from mycoplasma contamination prior to use.

For the assay, RAW 264.7 cells were seeded into 96‐well plates at a density of 1 × 10^5^ cells/well and incubated at 37°C in a CO_2_ incubator for 24 h. After incubation, the culture medium was aspirated. LPS (2 μg/mL) was added at a volume of 100 μL per well to the control and sample wells, while RPMI‐1640 medium alone was added to the blank wells. Then, 100 μL of the herbal extract solution was added to the sample and sample blank wells, whereas RPMI medium was added to the control and control blank wells. The cells were then incubated again at 37°C for 24 h. Following treatment, 100 μL of supernatant from each well was transferred to a new 96‐well plate. An equal volume (100 μL) of Griess reagent was added to each well, and the absorbance was measured at 570 nm using a microplate reader to determine NO concentration. The remaining supernatant in the original culture plate was then used for cytotoxicity assessment. 10 μL of MTT solution was added to each well, followed by incubation at 37°C for 2 h. The supernatant was then removed, and 100 μL of isopropanol in 0.04 M HCl was added to dissolve the formazan crystals. The plate was shaken, and the absorbance was measured at 570 nm to assess cell viability. Only concentrations with cell viability above 70% were considered for NO inhibition analysis. The percentage inhibition of NO production was calculated using the following formula:

(2) % inhibition=Abscontrol−AbssampleAbscontrol×100.

The IC_50_ (the concentration that inhibits 50% of NO production) was subsequently calculated from the dose–response curve. Dexamethasone (1 μM) was used as the positive control for NO inhibition. All experiments were performed in five replicates to ensure accuracy and reproducibility.

#### 2.3.5. Cytotoxicity

The cytotoxicity test was conducted on human keratinocyte HaCaT cells using the standard MTT (3‐(4,5‐dimethylthiazol‐2‐yl)‐2,5‐diphenyltetrazolium bromide) assay. HaCaT cells were obtained from CLS Cell Lines Service GmbH (Eppelheim, Germany; CLS order code: 300493) and were routinely tested and confirmed to be free from mycoplasma contamination prior to use. The cells were cultured in Dulbecco’s modified Eagle medium (DMEM) (LOT: 11424006, Corning, USA) supplemented with 10% (v/v) FBS (LOT: 0001656649, Sigma, USA), 2 mM L‐glutamine (LOT: 13624004, Corning, USA), and 1% penicillin–streptomycin antibiotic solution (Double antibiotics, 100×) (LOT: GA2403019, Servicebio, Wuhan, Hubei, China). The cells were maintained in a humidified incubator (Shel Lab, USA) at 37°C under 5% CO_2_ atmosphere until reaching approximately 95% confluency. The MTT assay was performed following a standard protocol to determine the half‐MIC (IC_50_) of the test samples. HaCaT cells were seeded in 96‐well plates at a density of 1 × 10^4^ cells/well and incubated for 24 h. The culture medium was then replaced with freshly prepared medium containing varying concentrations (15.625 μg/mL–1000 μg/mL) of *Centella asiatica* leaf extract, *Garcinia mangostana* pericarp extract, and *Chromolaena odorata* leaf extract, and incubated for another 24 h. Following treatment, 10 μL of MTT solution (10 mg/mL in phosphate‐buffered saline; PBS, LOT: 17124008, Corning, USA) was added to each well and incubated at 37°C for 3 h. After incubation, 100 μL of DMSO (LOT: K55418843336, Merck, Darmstadt, Germany) was added to each well to dissolve the resulting formazan crystals. The absorbance was measured at 570 nm using a microplate reader (FLUOstar Omega, Germany). The percentage of cell viability was calculated based on the optical density (OD) values. All experiments were performed in five replicates to ensure statistical reliability and reproducibility.

#### 2.3.6. Blood Clotting Ability

The coagulation activity of *Centella asiatica* leaf extract, *Garcinia mangostana* pericarp extract, and *Chromolaena odorata* leaf extract was evaluated using bovine blood. Fresh bovine blood, obtained as a by‐product from a commercial slaughterhouse in Chonburi Province, Thailand, was used for the assay. Platelets were isolated from whole blood through centrifugation at 400 revolutions per minute (rpm) for 10 min at room temperature. The supernatant was carefully removed, and the pellet at the bottom was subjected to a second round of centrifugation in 0.85% NaCl solution at 400 rpm for 10 min at room temperature. The supernatant was discarded, and this washing process with 0.85% NaCl solution was repeated until the supernatant became clear. The resulting platelet pellet was collected and stored at 4°C for a maximum of 5 days. To assess the coagulation‐inducing activity of the extracts, the platelet suspension obtained from centrifugation was diluted in normal saline at a 1 : 10 ratio. The MICs of *Centella asiatica*, *Garcinia mangostana*, and *Chromolaena odorata* extracts were each mixed with the diluted blood sample in a 1 : 1 (v/v) ratio. DMSO and normal saline were used as negative controls. The blood coagulation activity was then observed under a light microscope. For result interpretation, a drop of the mixture containing *Chromolaena odorata* extract and the diluted blood was examined microscopically. If blood aggregation or clumping was observed, it was considered an indication that the extract had coagulation‐inducing properties.

### 2.4. Preparation of Hydrogel Film

#### 2.4.1. Preliminary Study

To prepare the hydrogel film, PVA was weighed and dissolved in hot water at 80°C for 30 min. After complete dissolution, SCMC was added and stirred until the solution became homogeneous or until the polymer was fully swollen. Then, glycerin was added as a plasticizer and mixed thoroughly. The total weight of the solution was adjusted with distilled water to reach the desired formulation volume. The final solution was poured into a Petri dish and dried in an oven at 50°C for 24 h to form a film. The amounts of PVA and SCMC were varied according to Table 2 (F1–F9).

**TABLE 2:** Composition of hydrogel film formulations in the preliminary study.

Formula	PVA (g)	SCMC (g)	Glycerin (g)	DI water (mL)
F1	3.2	0.5	5	91.3
F2	3.2	1	5	90.8
F3	3.2	2	5	89.8
F4	4.8	0.5	5	89.7
F5	4.8	1	5	89.2
F6	4.8	2	5	88.2
F7	5.6	0.5	5	88.9
F8	5.6	1	5	88.4
F9	5.6	2	5	87.4
**F10**	**2**	**2**	**2**	**94**
**F11**	**3.34**	**0.67**	**2**	**94**
**F12**	**3.6**	**0.4**	**2**	**94**
**F13**	**2.5**	**2.5**	**2**	**93**
**F14**	**4.16**	**0.84**	**2**	**93**
**F15**	**4.5**	**0.5**	**2**	**93**
**F16**	**3**	**3**	**2**	**92**
**F17**	**5**	**1**	**2**	**92**
**F18**	**5.4**	**0.6**	**2**	**92**

*Note:* Rows highlighted in bold represent the experimental runs conducted under a 2‐factor, 3‐level full factorial design.

#### 2.4.2. Design of Experiment (DOE)

Following the identification of the experimental design space through preliminary studies, a full factorial DOE was employed to investigate the relationship between formulation variables of hydrogel film and film properties. A 2‐factor, 3‐level full factorial design was constructed and executed according to the assistance of the Design‐Expert® software (Version 12, Stat‐Ease Inc., Minneapolis, MN, USA). This design was selected because it allows for the evaluation of both linear and quadratic effects, as well as interactions between factors, providing a comprehensive understanding of how formulation variables influence hydrogel performance while maintaining a manageable number of experimental runs. The critical factors influencing the film properties and the design space for the DOE were determined based on the results of the preliminary study, ensuring that the selected factor levels were relevant for optimization and accurate modeling of the hydrogel’s performance. The experimental matrix, including formulations F10 to F18, is presented in Tables 2 and 3, highlighting the systematic variation of factors and the corresponding outcomes on film characteristics. In all DOE formulations (F10–F18), the herbal extracts were incorporated at the following concentrations: *Garcinia mangostana* extract, 3.12 mg; *Centella asiatica* extract, 25 mg; and *Chromolaena odorata* extract, 5 mg. These concentrations were selected based on their optimized biological activity and cytocompatibility profiles to ensure therapeutic efficacy while maintaining skin safety.

**TABLE 3:** Full factorial design with two factors (total polymer weight and PVA : SCMC ratio) at three levels for formulations F10–F18.

Run	Formulation	*X*_1_, total polymer weight (PVA + SCMC) (g)	*X*_2_, polymer ratio PVA : SCMC
1	F10	4 (−1)	1 : 1 (−1)
2	F11	4 (−1)	5 : 1 (0)
3	F12	4 (−1)	9 : 1 (+1)
4	F13	5 (0)	1 : 1 (−1)
5	F14	5 (0)	5 : 1 (0)
6	F15	5 (0)	9 : 1 (+1)
7	F16	6 (+1)	1 : 1 (−1)
8	F17	6 (+1)	5 : 1 (0)
9	F18	6 (+1)	9 : 1 (+1)

### 2.5. Characterization of Hydrogel Film

#### 2.5.1. Physical Appearance

The physical appearance of the hydrogel films was evaluated based on surface characteristics, homogeneity, transparency, and the presence of air bubbles. Observations were recorded using a digital camera both before and after oven drying. In addition, the polymer solution was visually assessed prior to being poured into Petri dishes to examine its uniformity and consistency.

#### 2.5.2. Film Mechanical Properties

The mechanical properties of film formulations developed under the DOE approach (F10–F18) were assessed in terms of breaking strain (*Y*_1_). For sample preparation, the polymer solutions were poured into glass plates with dimensions of 5 × 15 × 3 cm, ensuring equal weight across all samples to maintain consistent film thickness. Once dried completely, the films were cut into uniform strips measuring 1 × 10 cm, with 6 replicates prepared for each formulation. To standardize the testing area, transparent adhesive tape was affixed to both ends of each strip (1 cm at the top and bottom), leaving an 8‐cm section exposed for analysis. The breaking strain of each film was then measured using a texture analyzer.

#### 2.5.3. Swelling Properties

The swelling behavior of film formulations developed under the DOE approach (F10–F18) was assessed to determine their capacity to absorb liquid and expand. This evaluation simulates the hydrogel film’s ability to absorb wound exudate during practical application, a critical attribute for hydrogel‐based drug delivery systems intended for wound healing. Standard‐sized film samples (2.5 cm × 2.5 cm) were prepared and weighed using a high‐precision analytical balance to record the initial dry weight (*W*_0_). Each film was then treated with 1 mL of distilled water and carefully applied to the surface. After 5 min of contact time, any excess water was gently blotted from the film, and the sample was immediately reweighed to determine the swollen weight (*W*_1_). The swelling percentage (*Y*_2_) was calculated using the following equation:

(3) % swelling=W1−W0W0×100.

#### 2.5.4. Moisture Content

The moisture content of hydrogel film formulations developed under the DOE approach (F10–F18) was evaluated to determine the residual water content, which is a critical parameter affecting the film’s stability, flexibility, and performance. Film samples (2.5 cm × 2.5 cm) were prepared and placed into a moisture analyzer. The initial weight of each film was recorded prior to analysis. The instrument then automatically determined the moisture content percent (*Y*_3_) through thermal drying and displayed the result upon completion of the measurement, along with the final (dried) weight of the film. Each formulation was tested in triplicate to ensure accuracy and reproducibility of the results.

### 2.6. Statistical Analysis

The data obtained from individual experiments were initially analyzed using descriptive statistics. For comparative analysis, one‐way analysis of variance (ANOVA) was conducted to assess differences among multiple groups, followed by the Student’s *t*‐test for pairwise comparisons to determine statistical significance.

## 3. Results and Discussion

### 3.1. Herbal Extract

Based on the extraction results in Table 4, *Garcinia mangostana* showed the highest yield at 28.03%, followed by *Centella asiatica* at 25.54%, while *Chromolaena odorata* produced the lowest yield at 10.47%. These differences likely reflect variations in the content of extractable phytochemicals such as polyphenols, flavonoids, and xanthones among the plant species. The relatively high yields of *Garcinia mangostana* and *Centella asiatica* suggest a greater abundance of bioactive compounds and better solubility under the extraction conditions used. In contrast, the lower yield from *Chromolaena odorata* may indicate a lower concentration of extractable constituents or higher fibrous content that inhibits solvent penetration.

**TABLE 4:** Extraction yield and quantification of bioactive compounds in the herbal extracts.

Herbal extraction	Extraction yield (%)	Bioactive compounds	Bioactive compounds content (%w/w)
*Centella asiatica*	25.54	Madecassoside	0.03 ± 0.01
		Asiaticoside	0.01 ± 0.00
		Madecassic acid	0.000018 ± 0.00
		Asiatic acid	0.000003 ± 0.00

*Garcinia mangostana*	28.03	Alpha‐mangostin	18.50 ± 0.01

*Chromolaena odorata*	10.47	Gallic acid	0.09 ± 0.00
		Quercetin	0.06 ± 0.00
		Apigenin	0.16 ± 0.00

### 3.2. Phytochemical Analysis

The *Centella asiatica* extract was found to be rich in triterpenoid saponins and their aglycones, which are key contributors to its well‐documented therapeutic efficacy. As shown in Table 4, madecassoside (0.03 ± 0.01% w/w) was the predominant compound, followed by asiaticoside (0.01 ± 0.00% w/w), both of which are glycosylated forms of the bioactive triterpenes. In contrast, the corresponding aglycones (madecassic acid and asiatic acid) were detected in extremely low concentrations (0.000018 ± 0.00% and 0.000003 ± 0.00% w/w, respectively). This indicates that typical extraction and preservation conditions effectively maintain glycosidic bond integrity, minimizing breakdown into aglycones via enzymatic or environmental hydrolysis [27]. This suggests a higher prevalence of glycosidic forms in the extract, which are known to contribute significantly to the plant’s pharmacological effects, particularly in promoting wound healing [27], neuroprotection [28], and anti‐inflammatory responses [29].

As reported in Table 4, the analysis of *Garcinia mangostana* extract revealed the presence of alpha‐mangostin at a remarkably high concentration (18.50 ± 0.01% w/w). Alpha‐mangostin, a xanthone derivative, is recognized for its strong antioxidant, antimicrobial, and anticancer properties. Its dominance as the primary bioactive compound highlights *Garcinia mangostana* as a potent source of pharmacologically active xanthones [30, 31]. The high concentration of this single compound may be advantageous for standardization and targeted therapeutic applications, especially in managing oxidative stress and microbial infections.

In *Chromolaena odorata*, three flavonoid and phenolic compounds, including apigenin, gallic acid, and quercetin, were identified and quantified [5]. As shown in Table 4, apigenin was the most abundant (0.16 ± 0.00% w/w), followed by gallic acid (0.09 ± 0.00% w/w) and quercetin (0.06 ± 0.00% w/w). These compounds are associated with a broad range of bioactivities, including anti‐inflammatory, antimicrobial, and antioxidant effects [32–35]. The diversity and notable concentrations of these phenolics support the potential of *Chromolaena odorata* as a valuable source of bioactive natural products, particularly in the development of formulations aimed at infection control and tissue repair.

### 3.3. Antibacterial Activity

The antimicrobial properties of three plant extracts were assessed using MIC and MBC assays, which are essential parameters for evaluating the potential of wound healing patches to suppress microbial proliferation during application. MIC values were determined by the macro broth dilution method, while MBC values were obtained by subculturing the turbid mixtures from the MIC assays onto fresh agar plates to evaluate bactericidal activity. Table 5 summarizes the antibacterial efficacy of the extracts from *Centella asiatica*, *Garcinia mangostana*, and *Chromolaena odorata* against *Staphylococcus aureus* (MRSA) and *Staphylococcus epidermidis*. Among the tested extracts, *Garcinia mangostana* exhibited the highest antibacterial activity, with MIC and MBC values of 7.8 μg/mL and 15.6 μg/mL, respectively, against both bacterial strains. These results indicate strong bacteriostatic and bactericidal effects, likely attributable to the presence of xanthones and other active phytochemicals in the pericarp of *Garcinia mangostana* [10]. In contrast, *Chromolaena odorata* demonstrates moderate antimicrobial activity, with MIC and MBC values of 250 μg/mL and 500 μg/mL. Though less potent than *Garcinia mangostana*, it still exhibits effective inhibition and killing of both bacteria due to the presence of a diverse array of bioactive compounds, including flavonoids, alkaloids, tannins, and terpenoids. These phytochemicals are known to work synergistically to disrupt bacterial cell walls and inhibit microbial growth [5, 36]. Meanwhile, *Centella asiatica* shows MIC values greater than 4000 μg/mL for both bacteria, with MBC values undetermined, indicating negligible or no antibacterial activity under the conditions tested. This might be because the primary therapeutic benefits of *Centella asiatica* in wound healing are attributed more to its stimulation of collagen synthesis and anti‐inflammatory effects rather than direct, potent antibacterial action [13]. Furthermore, the concentration of its antibacterial compounds, such as triterpenoids, in the crude extract may have been too low to exhibit a significant inhibitory effect in this assay. The potent antibacterial properties of *Garcinia mangostana* and moderate activity of *Chromolaena odorata* reduce microbial contamination within the dressing environment. When incorporated into the PVA/SCMC hydrogel, these extracts ensure the dressing itself functions as an antimicrobial barrier, minimizing infection risk and prolonging dressing wear time without external antiseptics [37].

**TABLE 5:** MIC and MBC of plant extracts against *Staphylococcus aureus* (MRSA) and *Staphylococcus epidermidis*.

Extracts	*Staphylococcus aureus* (MRSA)		*Staphylococcus epidermidis*	
	MIC (μg/mL)	MBC (μg/mL)	MIC (μg/mL)	MBC (μg/mL)
*Centella asiatica*	> 4000	—	> 4000	—
*Garcinia mangostana*	7.8	15.6	7.8	15.6
*Chromolaena odorata*	250	500	250	500

*Note:* —, not determined.

### 3.4. Antioxidant Activity

**FIGURE 1:**
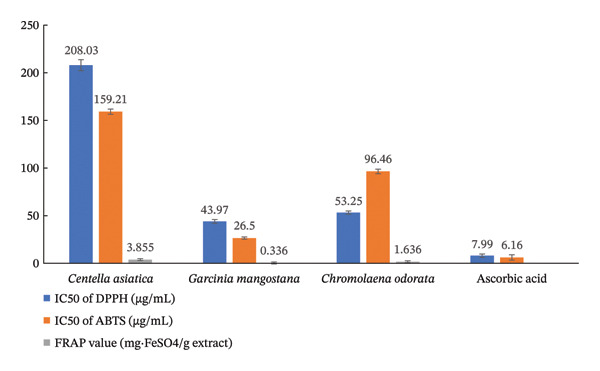
Comparative antioxidant activities of plant extracts and ascorbic acid measured by DPPH, ABTS, and FRAP assays.

The antioxidant activities of *Centella asiatica*, *Garcinia mangostana*, *Chromolaena odorata*, and ascorbic acid were evaluated using three complementary assays, which are DPPH and ABTS radical scavenging assays (expressed as IC_50_ values in μg/mL), and the FRAP assay (expressed in mg FeSO_4_/g extract). The results are summarized in Figure 1. Among the plant extracts, *Garcinia mangostana* exhibited the strongest antioxidant activity, with low IC_50_ values in both the DPPH (43.97 μg/mL) and ABTS (26.5 μg/mL) assays, indicating a high free radical scavenging potential. This was followed by *Chromolaena odorata* (DPPH: 53.25 μg/mL; ABTS: 96.46 μg/mL), while *Centella asiatica* showed the weakest activity with IC_50_ values of 208.03 μg/mL and 159.21 μg/mL for DPPH and ABTS, respectively. As expected, ascorbic acid, the positive control, displayed the highest antioxidant capacity with significantly lower IC_50_ values for DPPH (7.99 μg/mL) and ABTS (6.16 μg/mL), highlighting its superior radical scavenging efficiency. The FRAP assay results further supported these findings. *Garcinia mangostana* again demonstrated the highest ferric reducing power (0.336 mg FeSO_4_/g extract), followed by *Chromolaena odorata* (1.636 mg FeSO_4_/g), while *Centella asiatica* had a comparatively lower reducing capacity (3.855 mg FeSO_4_/g). Interestingly, despite *Centella asiatica*’s known bioactive profile, its antioxidant potency appears to be relatively modest based on these assays, possibly due to the low concentration of aglycone triterpenoids, which are generally more active antioxidants as discussed on the section of phytochemical analysis. These results collectively suggest that while *Centella asiatica* contains therapeutic compounds such as triterpenoid saponins, its overall antioxidant capacity is lower than that of *Garcinia mangostana* and *Chromolaena odorata*. The stronger antioxidant performance of *Garcinia mangostana* may be attributed to its rich content of xanthones, which are known for their high radical scavenging potential [38]. The strong antioxidant activity of *Garcinia mangostana* and *Chromolaena odorata* contributes to reducing oxidative stress at the wound site, which supports fibroblast survival and tissue regeneration [39].

### 3.5. Anti‐Inflammatory Activity

The anti‐inflammatory activity of *Chromolaena odorata*, *Garcinia mangostana*, and *Centella asiatica* extracts was evaluated by measuring NO production in LPS‐stimulated RAW 264.7 macrophage cells using the Griess‐modified assay. All three extracts demonstrated notable NO inhibition, indicating anti‐inflammatory potential. *Garcinia mangostana* showed the highest potency, achieving 83.5% NO reduction at a low concentration of 6.25 μg/mL, likely due to the presence of bioactive xanthones. *Chromolaena odorata* also exhibited strong activity, reducing NO by 79.4% at 50 μg/mL. In contrast, *Centella asiatica* required a much higher concentration (500 μg/mL) to achieve the highest NO inhibition (95.5%), suggesting that while its active constituents are effective, they are less potent and require greater concentrations for comparable effects. The observed results provide a clear rationale for the combined use of these three extracts in a wound healing formulation, as each contributes uniquely to managing inflammation. The exceptional potency of *Garcinia mangostana* at a mere 6.25 μg/mL underscores the well‐documented ability of its constituent xanthones, particularly alpha‐mangostin, to potently suppress proinflammatory pathways like NF‐κB, which directly controls the expression of the iNOS enzyme responsible for producing NO [40, 41]. *Chromolaena odorata*, containing flavonoids and terpenoids, complements this by providing strong, broad‐spectrum anti‐inflammatory action at a still moderate concentration [42, 43]. The most telling result is that of *Centella asiatica*, which required a significantly higher concentration to inhibit NO. This does not indicate a lack of utility but rather highlights its different, yet complementary, therapeutic role. The primary wound healing benefits of *Centella asiatica* are famously linked to its triterpenoids stimulating collagen synthesis and promoting tissue remodeling functions that are distinct from potent, initial‐phase inflammation suppression [44]. Therefore, a combination of these extracts appears highly strategic: *Garcinia mangostana* and *Chromolaena odorata* can act as the primary, fast‐acting agents to rapidly reduce acute inflammation and microbial threats in the early stages of wound care, while *Centella asiatica*, although less potent in this specific assay, would exert its well‐established regenerative and modulatory effects to support the subsequent proliferative and remodeling phases of healing. Moreover, the hydrogel’s sustained‐release behavior allows gradual diffusion of these anti‐inflammatory constituents, ensuring prolonged therapeutic action at the wound interface [45].

### 3.6. Cytotoxicity

**FIGURE 2:**
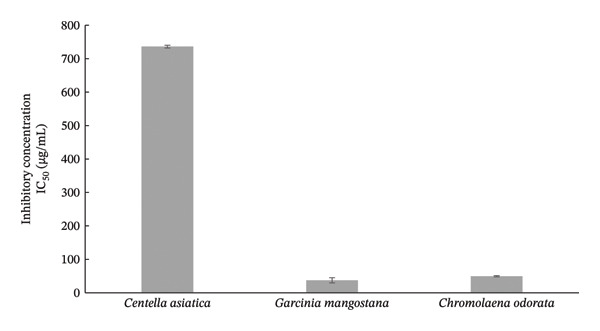
Cytotoxicity of plant extracts on HaCaT cells assessed by MTT assay.

The cytotoxicity of *Centella asiatica*, *Garcinia mangostana*, and *Chromolaena odorata* extracts was evaluated using the MTT assay on HaCaT keratinocyte cells, with IC_50_ values calculated to determine the concentration at which 50% of the cells are inhibited. This test is crucial for assessing the safety of plant‐derived compounds intended for topical use, particularly in wound healing applications, where minimizing cytotoxic effects on skin cells is essential. The results show a clear distinction in the safety profiles of the three extracts. *Centella asiatica* exhibited the highest IC_50_ value (736.11 ± 4.08 μg/mL), classifying it as low toxic based on Clarkson’s toxicity criterion (IC_50_ between 500 and 1000 μg/mL). This suggests that *Centella asiatica* is biocompatible and safe for use on skin cells, supporting its traditional use in wound healing and skin regeneration [46]. In contrast, *Garcinia mangostana* and *Chromolaena odorata* had significantly lower IC_50_ values (37.42 ± 7.80 μg/mL and 49.55 ± 1.85 μg/mL, respectively), placing them in the highly toxic category (IC_50_ < 100 μg/mL) (Figure 2). Despite their strong antibacterial properties (as previously shown), these results raise concerns regarding their potential cytotoxicity toward human skin cells. Therefore, the critical path forward is to optimize the concentrations within the final herbal cocktail to establish a therapeutic window. This will ensure the formulation leverages the potent antibacterial activity of *Garcinia mangostana* and *Chromolaena odorata* at levels that remain safely below their cytotoxic thresholds for keratinocytes, thereby balancing antimicrobial efficacy with cellular safety at the wound site. Moreover, their application in wound care formulations may therefore require concentration adjustments, encapsulation strategies, or formulation with biocompatible carriers to mitigate cellular damage. In summary, while *Garcinia mangostana* and *Chromolaena odorata* are promising for antimicrobial effects, their use must be balanced with cytotoxicity considerations. *Centella asiatica*, though less effective against bacteria, presents a safer profile for skin application, making it more suitable for direct inclusion in wound healing systems.

The formulation of the hydrogel and the use of a mixed herbal strategy can effectively mitigate the observed cytotoxicity of *Garcinia mangostana* and *Chromolaena odorata* extracts. The incorporation of *Centella asiatica* plays a pivotal role in enhancing overall cytocompatibility, as its biocompatible triterpenoids help buffer the cytotoxic potential of the more potent extracts through synergistic dilution, thereby maintaining therapeutic efficacy while minimizing adverse effects. During hydrogel preparation, the concentrations of *Garcinia mangostana* and *Chromolaena odorata* were optimized to remain below cytotoxic thresholds, ensuring antimicrobial and anti‐inflammatory efficacy without compromising cell viability [47]. Moreover, the sustained‐release profile of the hydrogel provides gradual and localized delivery of active compounds, reducing peak exposure at the wound interface. This can limit direct cellular exposure to high concentrations of the extracts and thereby enhance the overall biocompatibility of the formulation [48]. Future improvements may involve encapsulating the extracts within polymeric micelles, nanoparticles, or liposomal carriers to modulate release kinetics, prevent burst release, and control diffusion spatially at the wound site [49].

### 3.7. Blood Clotting Ability

**FIGURE 3:**
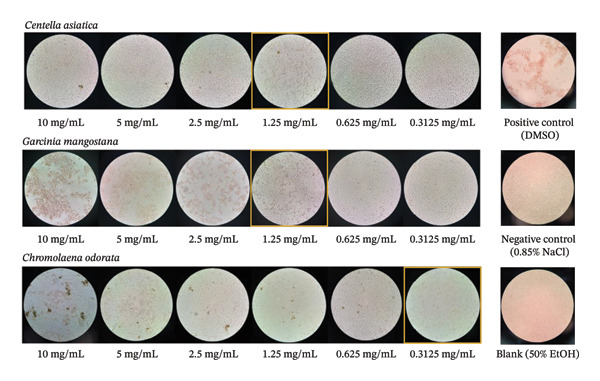
Microscopic images demonstrating the blood clotting activity of *Centella asiatica*, *Garcinia mangostana*, and *Chromolaena odorata* extracts at various concentrations.

The hemostatic potential of the plant extracts was evaluated by observing their ability to induce the aggregation of bovine platelets *in vitro*, a critical first step in the wound healing cascade. The results of the microscopic blood clotting assay are presented in Figure 3. The controls performed as expected: the positive control (DMSO) induced significant cellular aggregation, while the negative control (0.85% NaCl) and the blank solvent control (50% EtOH) showed a uniform, nonaggregated suspension of cells, establishing a clear baseline for interpretation. All three plant extracts demonstrated dose‐dependent procoagulant activity. *Chromolaena odorata*, a plant traditionally renowned for its styptic properties [50], induced visible platelet starting aggregation at concentrations of 0.3125 mg/mL and more strong and visible aggregate at 5 and 10 mg/mL. Moreover, both *Garcinia mangostana* and *Centella asiatica* also exhibited potent hemostatic effects. *Garcinia mangostana* pericarp extract caused strong platelet aggregation, with a clear effect observed down to a concentration of 1.25 mg/mL. Similarly, *Centella asiatica* extract also effectively induced clotting, showing visible aggregation down to the same concentration threshold of 1.25 mg/mL, although the aggregates were less dense compared to those from *Garcinia mangostana*. These visual results confirm that all three extracts possess procoagulant properties, which are essential for achieving hemostasis in a wound environment.

The findings from this study provide valuable insight into the comprehensive wound healing capabilities of the selected extracts. The strong procoagulant effect of *Chromolaena odorata* aligns with its widespread traditional use for stopping bleeding. Its mechanism is likely attributable to its rich content of flavonoids and tannins, which are known to promote hemostasis by activating the coagulation cascade and facilitating platelet plug formation. The significant clotting activity induced by the *Garcinia mangostana* pericarp extract is a noteworthy finding. While primarily studied for its antibacterial and anti‐inflammatory xanthones, the pericarp is also rich in tannins, which are potent protein‐precipitating and cell‐aggregating agents. This suggests that *Garcinia mangostana* can contribute dually to wound healing by not only preventing infection but also aiding in the initial hemostasis phase. Perhaps most interestingly, *Centella asiatica*, which is celebrated for its role in the later, proliferative phase of wound healing (i.e., stimulating collagen synthesis and tissue remodeling), also demonstrated considerable hemostatic potential. This suggests that *Centella asiatica* may offer a broader therapeutic benefit than commonly acknowledged, contributing to the very first stage of wound repair in addition to its effects on tissue regeneration. Therefore, the combination of these three extracts in a single wound healing application is highly strategic. Together, they can provide a multipronged therapeutic action: rapid hemostasis to control bleeding (*Chromolaena odorata* and *Garcinia mangostana*), coupled with the essential tissue regenerative properties of *Centella asiatica*, creating a comprehensive formulation that addresses multiple critical stages of the wound healing process. Furthermore, when utilized in a practical application, the hydrogel’s notable liquid absorption capacity, synergistically combined with the extract’s hemostatic activity, ensures the immediate control of bleeding upon deployment.

### 3.8. Preparation of Hydrogel Film

The physical characteristics of film formulations F1–F9 were preliminary evaluated based on clarity, viscosity, bubble formation, and postoven film texture. All formulations appeared clear and homogeneous, indicating good miscibility of components. Bubble formation was minimal in most samples, except in those containing higher concentrations of SCMC (F3, F6, F9), where slight bubbles persisted even after 24 h. Notably, formulations with lower SCMC content (F1, F4, F7) remained bubble‐free throughout the observation period, suggesting improved stability at lower viscosity levels. As SCMC concentration increased from 0.5 to 2 g across each PVA group (40%, 60%, and 70%), there was a corresponding increase in viscosity and bubble formation [51]. This effect was most evident in formulations F3, F6, and F9, which exhibited relatively higher viscosity and retained slight surface foam after overnight storage. The translucency observed in F3 compared to F1 and F2 further supports the influence of SCMC on optical clarity, likely due to increased molecular entanglement and scattering.

In terms of film characteristics after drying in a hot air oven, all formulations produced smooth, homogeneous films. However, notable differences were observed in texture. F5, containing 60% PVA and 1% SCMC, yielded a film that was clear, stretchable, and nonbrittle properties ideal for applications requiring flexibility, such as wound dressings or transdermal patches. In contrast, F7 and F8 (70% PVA) exhibited an oily surface texture, possibly due to incomplete glycerin absorption in the highly viscous matrix, which could influence the user experience and film adhesion [52]. After the preliminary study was completed, the experimental design space was established, as presented in Tables 2 and 3 (Formulations F10–F18).

### 3.9. Design of Experiment (DOE)

#### 3.9.1. Breaking Strain (*Y*_1_)

Breaking strain reveals how the polymer ratio (PVA : SCMC) and total polymer weight (PVA + SCMC) influence the mechanical strength of hydrogel films. The plot in Figure 4(a) shows a nonlinear interaction between these two factors. As the total polymer weight increases, breaking strain also tends to increase, particularly when the polymer ratio is balanced or slightly PVA‐rich. This suggests that a denser polymer network contributes to improved tensile properties, as more crosslinking sites and film thickness help resist tearing. However, at extremely high PVA ratios and lower total weights, the strain drops, likely due to excessive rigidity and brittleness introduced by too much PVA, which lacks the flexibility that SCMC contributes.

FIGURE 43D response surface plots showing the effects of polymer ratio (PVA : SCMC) and total polymer weight (PVA + SCMC) on key film properties: (a) Breaking strain, (b) swelling percentage, and (c) moisture content percentage.

**Figure:**
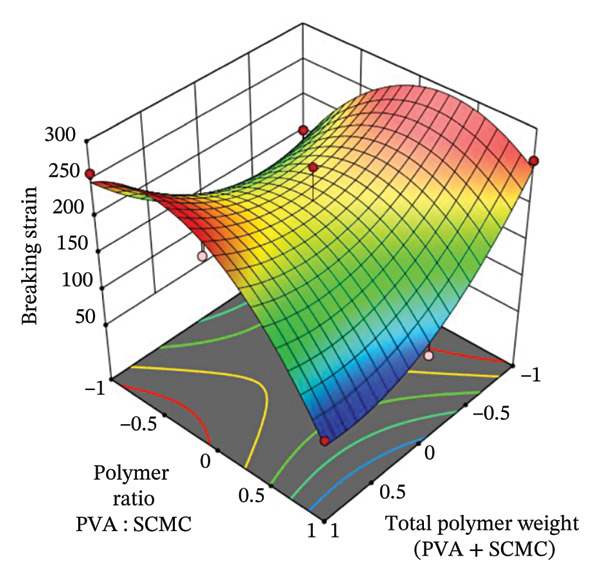
(a)

**Figure:**
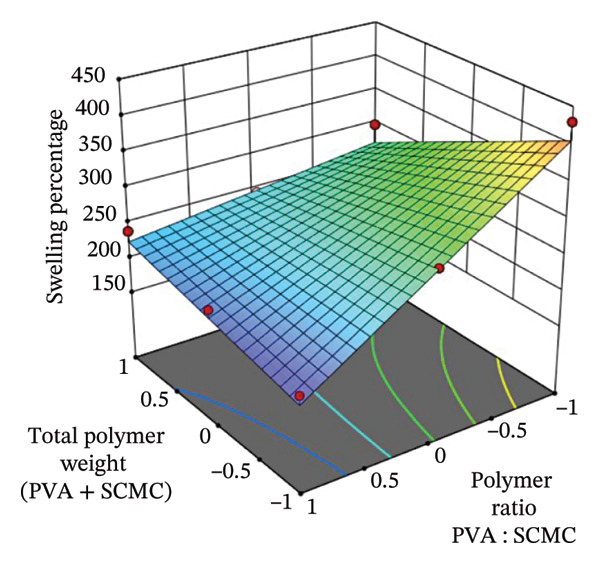
(b)

**Figure:**
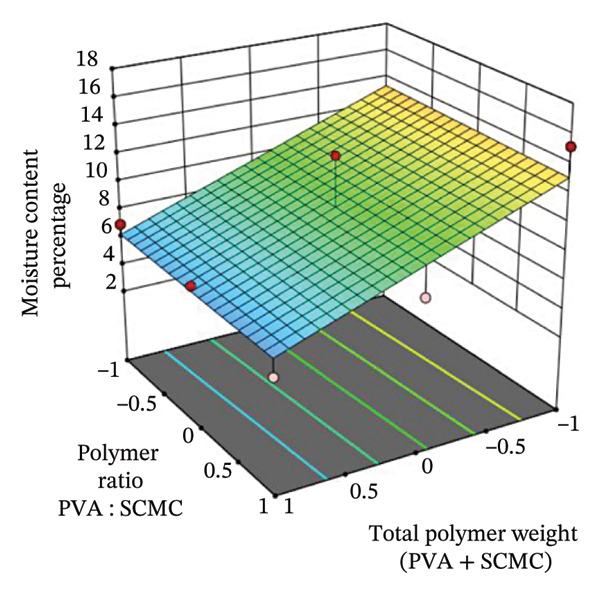
(c)

This result can also be explained by the partial least squares (PLS) model of *Y*_1_ in Table 6, as further illustrated by the corresponding 3D response surface plot. The model identifies significant interaction and quadratic effects, particularly from *X*_2_ (total polymer weight). Increasing the total polymer weight (*X*_2_) enhances breaking strain up to an optimal point, after which excessive polymer loading (indicated by the large negative quadratic term −91.26$X_{2}^{2}$) reduces film flexibility and may introduce brittleness. The negative coefficients for both *X*_1_ and *X*_2_ also imply that high values alone do not directly improve strength, emphasizing the importance of balanced composition. Statistically, the model exhibits a high coefficient of determination (*R* ^2^ = 0.9104), indicating strong predictive power and a good fit to the experimental data. While the *p* value (0.0838) is slightly above the conventional threshold of 0.05, it still suggests a moderate level of significance, especially in the context of multivariable interaction modeling. The *F* value of 6.10 supports the relevance of the model in explaining variance in breaking strain across the tested formulations.

**TABLE 6:** PLS regression equations with *p* values, *F* values, and *R* ^2^ for hydrogel film responses: breaking strain (*Y*_1_), swelling percentage (*Y*_2_), and moisture content (*Y*_3_).

Responsible variables	Unit	PLS models (code equations)	*p* value	*F* value	*R* ^2^
Breaking strain (*Y*_1_)	—	*Y*_1_ = −15.02*X*_1_ − 14.60*X*_2_ − 70.24*X*_1_ *X*_2_ + 53.94*X*_2_ − 91.26$X_{2}^{2}$	0.0838	6.10	0.9104
Swelling percentage (*Y*_2_)	%	*Y*_2_ = +267.50 − 20.60*X*_1_ − 70.27*X*_2_ + 46.76*X*_1_ *X*_2_	0.0214	8.39	0.8342
Moisture content percentage (*Y*_3_)	%	*Y*_3_ = 9.66 − 3.57*X*_1_ − 0.1367*X*_2_	0.0193	8.18	0.7316

To explain this phenomenon at the molecular level, the total polymer weight (*X*_2_) increases, breaking strain also tends to increase, particularly when the polymer ratio is balanced or slightly PVA‐rich. This suggests that a denser polymer network contributes to improved tensile properties, as more crosslinking sites and film thickness help resist tearing. Mechanistically, a higher concentration of both PVA and SCMC increases the probability of physical crosslinking, primarily through secondary forces like hydrogen bonding. This enhanced molecular entanglement effectively increases the cohesive strength of the hydrogel network, thus requiring more energy to rupture the film (higher tensile strength and breaking strain) [53, 54]. However, at extremely high PVA ratios and lower total weights, the strain drops, likely due to excessive rigidity and brittleness. This reduction in flexibility at high PVA content is molecularly attributed to the high degree of crystallinity and strong interchain hydrogen bonding inherent to PVA, making the polymer network stiffer and more brittle [55]. The inclusion of SCMC acts as a network modifier, disrupting the tight packing of PVA chains, which, along with the plasticizing effect of glycerin, imparts the necessary chain mobility and flexibility for higher strain values. Altogether, this modeling confirms that both polymer ratio and weight must be finely tuned to achieve hydrogel films with optimal mechanical integrity, crucial for practical applications such as wound dressings where both flexibility and tensile strength are required.

#### 3.9.2. Swelling Percentage (*Y*_2_)

The 3D response surface plot for swelling percentage (*Y*_2_) in Figure 4(b) illustrates the influence of polymer ratio (*X*_1_: PVA : SCMC) and total polymer weight (*X*_2_: PVA + SCMC) on the water absorption behavior of hydrogel films. The response surface shows a pronounced slope upward toward high SCMC content and high total polymer weight, indicating that these conditions significantly enhance the swelling capacity of the films. This massive water uptake is primarily driven by the anionic carboxylate groups (‐COO^−^) along the SCMC chain. These highly hydrophilic and charged groups draw in large amounts of water through osmotic pressure and bind it efficiently via hydrogen bonding. The mutual electrostatic repulsion between adjacent negative charges also forces the polymer network to expand, maximizing the swelling capacity (swelling ratio) [56, 57]. Conversely, increasing the proportion of PVA or reducing the overall polymer content leads to a lower swelling response. Although PVA is also hydrophilic, excessive PVA or high total polymer weight results in a higher cross‐link density (tighter physical network). This dense, restrictive structure physically impedes the influx of water and limits the space for chain expansion, thereby inhibiting the overall swelling ratio [58, 59]. These experimental observations are quantitatively supported by the PLS regression model for swelling percentage (Table 6): *Y*_2_ = +267.50 − 20.60*X*_1_ − 70.27*X*_2_ + 46.76*X*_1_ *X*_2_, which reveals a strong positive interaction between polymer ratio and weight, as seen in the interaction term (+46.76*X*_1_ *X*_2_). Although the linear coefficients for both *X*_1_ and *X*_2_ are negative, the positive interaction term offsets their individual inhibitory effects when optimized together. The model’s *p* value (0.0214) and *F* value (8.39) indicate statistically significant effects, while an *R* ^2^ value of 0.8342 confirms good model fit and predictability. Altogether, these results suggest that maximizing swelling (crucial for wound exudate absorption) requires a strategic combination of higher SCMC content and adequate total polymer loading, avoiding over‐crosslinking that could hinder water uptake.

#### 3.9.3. Moisture Content Percentage (*Y*_3_)

The influence of the polymer ratio (PVA : SCMC) and total polymer weight on the moisture content of the hydrogel films is depicted in the 3D response surface plot (Figure 4(c)). The graph reveals a relatively linear relationship where the moisture content percentage is influenced by both factors. A clear trend is visible along the polymer ratio axis; as the ratio shifts from being PVA‐dominant (coded level −1) to SCMC‐dominant (coded level 1), the moisture content percentage decreases significantly. This seemingly counterintuitive result is due to SCMC’s superior capability for strong water binding (high affinity for water). While SCMC absorbs more water during the formulation, its strong chemical interaction with water allows it to hold that water more tightly, leading to less evaporative loss during the oven drying process and a lower residual moisture content in the final dried film. Lower moisture content in SCMC‐rich films reflects stronger water immobilization [60]. Similarly, although less pronounced, an increase in the total polymer weight also appears to contribute to a lower moisture content. The highest moisture content is observed at low levels of total polymer weight and a high proportion of PVA. This is because PVA and the plasticizer, glycerin, primarily create hydrophilic domains that hold “free water” less tightly than the highly charged SCMC polymer. This weakly bound water is more readily retained and measured as residual moisture after the drying step, making the PVA‐dominant formulas appear to have higher moisture content [53, 61]. The planar nature of the response surface suggests that there are no significant interactive or quadratic effects between the two variables on the film’s final moisture content. From the statistical analysis presented in Table 6, the relationship is quantified by the PLS regression model for moisture content (*Y*_3_): *Y*_3_ = 9.66 − 3.57*X*_1_ − 0.1367*X*_2_. This model confirms the observations from the 3D plot, with the negative coefficients for both polymer ratio (−3.57*X*_1_) and total polymer weight (−0.1367*X*_2_), indicating that increasing either factor leads to a decrease in moisture content. The much larger coefficient for *X*_1_ (polymer ratio) highlights it as the more influential factor. The model is statistically significant, with a *p* value of 0.0193 (which is less than 0.05) and an *F* value of 8.18. The coefficient of determination (*R* ^2^) is 0.7316, signifying that approximately 73.2% of the variability in moisture content can be explained by this linear model. The results imply that the amount of residual water in the film, a critical parameter for stability and flexibility, is strongly dictated by the polymer composition, with higher SCMC content leading to drier films.

#### 3.9.4. Optimization

Upon completion of the experiments designed via DOE, a numerical optimization was conducted to determine the optimal formulation. The criteria were established to achieve specific, desirable material properties as shown in Table 7: maximizing the breaking strain (*Y*_1_), maximizing the swelling percentage (*Y*_2_), and constraining the moisture content (*Y*_3_) to a target range of 4.70%–15.17%. This is because a high breaking strain is critical for ensuring the material is flexible, durable, and can withstand mechanical stress without premature failure during application. Maximizing the swelling percentage is desirable for applications requiring high absorbency, such as wound exudate management or controlled drug release. However, moisture content must be carefully controlled; excessively high levels can compromise mechanical integrity and create an environment susceptible to microbial growth, while insufficient moisture can lead to brittleness and poor flexibility. The optimal hydrogel film formulation, as determined by PLS models, consists of a total polymer weight (*X*_1_) of 4.0 g and a PVA‐to‐SCMC mass ratio (*X*_2_) of 2.434 : 1. This formulation yields the most favorable balance of the targeted properties, with an overall desirability score of 0.757.

**TABLE 7:** Comparison of experimental and predicted response values for the optimized formulation.

Responses	Optimized target goal	Actual experimental value	Calculated value of the PLS models	% Prediction error
Breaking strain (*Y*_1_)	Maximize	258.89	248.20	4.13%
Swelling percentage (*Y*_2_)	Maximize	256.86%	333.88%	29.99%
Moisture content percentage (*Y*_3_)	In range	14.58%	13.28%	8.92%

To validate the accuracy of the optimization, the predicted optimal hydrogel formulation was prepared and its properties were experimentally determined. A comparison between the actual experimental values (*Y*_1_, *Y*_2_, and *Y*_3_) and the theoretical values calculated by the PLS model is presented in Table 6. The experimental results show a strong correlation with the predicted values, confirming the validity and accuracy of the optimization model. The prediction errors for breaking strain (*Y*_1_), swelling percentage (*Y*_2_), and moisture content percentage (*Y*_3_) were found to be 4.13%, 29.99%, and 8.92%, respectively. These values indicate that the PLS model is highly accurate for predicting breaking strain and moisture content, with relatively low deviations between experimental and predicted outcomes. The higher error observed in the swelling percentage may be attributed to greater sensitivity of this parameter to experimental variations or unmodeled interactions between components affecting hydrogel behavior. Despite this, the swelling value achieved experimentally (256.86%) remains high and functionally suitable for absorbent applications. The overall consistency between predicted and observed values supports the robustness of the PLS optimization approach and affirms its utility in guiding the development of hydrogel systems tailored for biomedical or pharmaceutical applications.

In the optimized hydrogel formulation, the concentrations of *Garcinia mangostana* (31.2 μg/mL), *Centella asiatica* (250 μg/mL), and *Chromolaena odorata* (50 μg/mL) were maintained well below their respective IC_50_ cytotoxicity values, thereby ensuring safety while preserving biological activity. These concentrations were sufficient to retain the antioxidant, antibacterial, and anti‐inflammatory functions of the extracts, which collectively enhance wound healing potential through infection control, oxidative stress reduction, and tissue regeneration. Moreover, the PVA/SCMC hydrogel matrix serves as a diffusion‐controlling carrier, promoting sustained and localized release of bioactive compounds, thus minimizing direct cellular exposure to high concentrations and further improving cytocompatibility. Based on these compositional and release characteristics, the final composite hydrogel can be considered safe and effective for topical wound applications. Nevertheless, direct cytotoxicity and irritation testing of the complete hydrogel film are planned for future studies to experimentally confirm its safety profile.

The present study offers a distinctive contribution to the field of herbal‐based wound care through the development of a tri‐herbal hydrogel system that integrates *Garcinia mangostana*, *Centella asiatica*, and *Chromolaena odorata* within an optimized PVA/SCMC polymeric matrix. Unlike previous formulations that employed single or dual plant extracts (Table 1), this work combines three herbal agents with complementary bioactivities, antibacterial, anti‐inflammatory, antioxidant, and hemostatic, resulting in a multifunctional dressing capable of addressing multiple phases of the wound healing process. The application of a DOE approach further enhances the scientific rigor of formulation optimization, ensuring an ideal balance between mechanical strength, swelling capacity, and biocompatibility. This integration of phytotherapy with material science demonstrates a novel, holistic approach to wound management and establishes a foundation for next‐generation, evidence‐based herbal wound dressings with potential translational value for chronic and infected wounds.

## 4. Conclusion

This study successfully developed and optimized a multifunctional herbal hydrogel film incorporating *Garcinia mangostana*, *Centella asiatica*, and *Chromolaena odorata* extracts for advanced wound dressing applications. Each extract contributes distinct yet complementary pharmacological effects, *Garcinia mangostana* provides potent antibacterial and antioxidant properties, *Centella asiatica* enhances biocompatibility and tissue regeneration, and *Chromolaena odorata* offers strong anti‐inflammatory and hemostatic actions. The synergistic integration of these extracts enables the hydrogel to effectively address multiple stages of wound healing, including infection control, inflammation reduction, and tissue repair. The optimized PVA/SCMC hydrogel matrix, established through a DOE approach, demonstrated excellent mechanical integrity, swelling capacity, and appropriate moisture content, ensuring practical functionality for wound care. From a clinical perspective, this herbal‐based hydrogel film represents a safe, natural, and cost‐effective alternative to conventional dressings, with high potential for future translational use in chronic and infected wound management.

Further studies on the pharmacological activities and toxicity of the herbal mixture in the form of extract and hydrogel film formulations at different extract concentrations are warranted to draw more comprehensive conclusions. Moreover, future work should focus on evaluating the long‐term stability, release kinetics, and scalability of the optimized hydrogel formulation. Investigating its biocompatibility and healing performance in suitable animal models, followed by well‐designed clinical trials, will be crucial to validate its therapeutic efficacy and ensure safety in real‐world wound care applications.

## Author Contributions

Conceptualization, Tanikan Sangnim and Kampanart Huanbutta; methodology, Chonlada Panpipat, Suwisit Manmuan, Nontanat Leehueng, Wasutthanat Suphan, and Chanapa Thuenaram; formal analysis, Tanikan Sangnim and Kampanart Huanbutta; investigation, Chonlada Panpipat, Suwisit Manmuan, Nontanat Leehueng, Wasutthanat Suphan and Chanapa Thuenaram; data curation, Chonlada Panpipat and Kampanart Huanbutta; writing–original draft preparation, Tanikan Sangnim, Chonlada Panpipat, Nontanat Leehueng, Wasutthanat Suphan, and Chanapa Thuenaram; writing–review and editing, Kampanart Huanbutta; visualization, Kampanart Huanbutta; supervision, Tanikan Sangnim; project administration, Kampanart Huanbutta; funding acquisition, Kampanart Huanbutta.

## Funding

This research was funded by Rangsit University, Grant No. 52/2566.

## Disclosure

All authors have read and agree to the published version of the manuscript.

## Ethics Statement

Ethical approval was not required for this study. All cell lines used in the experiments (HaCaT human keratinocytes and RAW 264.7 murine macrophages) were commercially obtained from certified cell repositories (CLS Cell Lines Service GmbH and the American Type Culture Collection, ATCC) and were not derived directly from human participants or experimental animals by the authors. The blood samples used for the blood clotting assay were bovine blood obtained as a by‐product from routine slaughtering processes for food production at a licensed commercial slaughterhouse. No live animals were used or sacrificed specifically for research purposes. Therefore, this study did not involve human subjects or experimental animals and did not fall under the scope of institutional or national ethics committee approval requirements.

## Conflicts of Interest

The authors declare no conflicts of interest.

## Data Availability

The data that support the findings of this study are available from the corresponding author upon reasonable request.

## References

[1] Percival N. J. , Classification of Wounds and Their Management, Surgery. (2002) 20, no. 5, 114–117, 10.1383/surg.20.5.114.14626.

[2] Harding K. , Morris H. , and Patel G. , Healing Chronic Wounds, BMJ. (2002) 324, no. 7330, 160–163. 11799036 10.1136/bmj.324.7330.160 PMC1122073

[3] Sen C. K. , Human Wounds and Its Burden: An Updated Compendium of Estimates, Advances in Wound Care. (2019) 8, no. 2, 39–48, 10.1089/wound.2019.0946, 2-s2.0-85061637585. 30809421 PMC6389759

[4] Boateng J. S. , Matthews K. H. , Stevens H. N. , and Eccleston G. M. , Wound Healing Dressings and Drug Delivery Systems: A Review, Journal of Pharmaceutical Sciences. (2008) 97, no. 8, 2892–2923, 10.1002/jps.21210, 2-s2.0-52449133379. 17963217

[5] Sangnim T. , Meeboon P. , Phongsewalak P. et al., Development and Evaluation of Liquid Plaster Loaded With *Chromolaena odorata* Leaf Extract Endowed With Several Beneficial Properties to Wound Healing, Gels. (2022) 8, no. 2, 10.3390/gels8020072. PMC8871034 35200454

[6] Sangnim T. , Puri V. , Dheer D. , Venkatesh D. N. , Huanbutta K. , and Sharma A. , Nanomaterials in the Wound Healing Process: New Insights and Advancements, Pharmaceutics. (2024) 16, no. 3, 10.3390/pharmaceutics16030300. PMC10976133 38543194

[7] Huanbutta K. , Sittikijyothin W. , and Sangnim T. , Development of Topical Natural Based Film Forming System Loaded Propolis From Stingless Bees for Wound Healing Application, Journal of Pharmaceutical Investigation. (2020) 50, no. 6, 625–634, 10.1007/s40005-020-00493-w.

[8] Nikam A. , Thomas A. , Giram P. , Nagore D. , and Chitlange S. , Herbal-Based Dressings in Wound Management, Current Diabetes Reviews. (2023) 19, no. 4, 46–58, 10.2174/1573399818666220401105256. 35366781

[9] Gokarneshan N. , Application of Natural Polymers and Herbal Extracts in Wound Management, Advanced Textiles for Wound Care, 2019, Elsevier, Amsterdam, 541–561.

[10] Semangoen T. , Chotigawin R. , Sangnim T. et al., Antimicrobial Efficacy of Mangosteen (*Garcinia mangostana*) Peel Extracts in Airborne Microbial Control Within Livestock Farming Environments, Microbial Pathogenesis. (2025) 204, 10.1016/j.micpath.2025.107618. 40254079

[11] Gondokesumo M. E. , Sumitro S. B. , Handono K. , Pardjianto B. , Widowati W. , and Utomo D. H. , A Computational Study to Predict Wound Healing Agents From the Peel of the Mangosteen (*Garcinia mangostana* L.) Extract, International Journal Bioautomation. (2020) 24, no. 3, 265–276, 10.7546/ijba.2020.24.3.000607.

[12] Bylka W. , Znajdek‐Awiżeń P. , Studzińska‐Sroka E. , Dańczak‐Pazdrowska A. , and Brzezińska M. , *Centella asiatica* in Dermatology: An Overview, Phytotherapy Research. (2014) 28, no. 8, 1117–1124, 10.1002/ptr.5110, 2-s2.0-84905658596. 24399761

[13] Arribas-López E. , Zand N. , Ojo O. , Snowden M. J. , and Kochhar T. , A Systematic Review of the Effect of *Centella asiatica* on Wound Healing, International Journal of Environmental Research and Public Health. (2022) 19, no. 6. 10.3390/ijerph19063266 PMC8956065 35328954

[14] Odutayo F. , Ezeamagu C. , Kabiawu T. , Aina D. , and Mensah-Agyei G. , Phytochemical Screening and Antimicrobial Activity of *Chromolaena odorata* Leaf Extract Against Selected Microorganisms, Journal of Advances in Medical and Pharmaceutical Sciences. (2017) 13, no. 4, 1–9, 10.9734/jamps/2017/33523.

[15] Pandith H. , Zhang X. , Liggett J. , Min K.-W. , Gritsanapan W. , and Baek S. J. , Hemostatic and Wound Healing Properties of *Chromolaena odorata* Leaf Extract, International Scholarly Research Notices. (2013) 2013, 168269–8, 10.1155/2013/168269. PMC3747403 23984087

[16] Phumlek K. , Itharat A. , Pongcharoen P. et al., *Garcinia mangostana* Hydrogel Patch: Bactericidal Activity and Clinical Safety for Acne Vulgaris Treatment, Research in Pharmaceutical Sciences. (2022) 17, no. 5, 457–467, 10.4103/1735-5362.355195. 36386483 PMC9661683

[17] Vamvanij N. , Chuangsuwanich A. , Charoonrut P. , and Cheunsuchon P. , Evaluation of Combined Herbal Extract Dressing Materials Effect on Open Wounds in Pig Model, Journal of the Medical Association of Thailand. (2017) 100.

[18] Panawes S. , Ekabutr P. , Niamlang P. , Pavasant P. , Chuysinuan P. , and Supaphol P. , Antimicrobial Mangosteen Extract Infused Alginate-Coated Gauze Wound Dressing, Journal of Drug Delivery Science and Technology. (2017) 41, 182–190, 10.1016/j.jddst.2017.06.021, 2-s2.0-85025104960.

[19] Qi L. , Zhang C. , Wang B. , Yin J. , and Yan S. , Progress in Hydrogels for Skin Wound Repair, Macromolecular Bioscience. (2022) 22, no. 7, 10.1002/mabi.202100475. 35388605

[20] Kuddushi M. , Shah A. A. , Ayranci C. , and Zhang X. , Recent Advances in Novel Materials and Techniques for Developing Transparent Wound Dressings, Journal of Materials Chemistry B. (2023) 11, no. 27, 6201–6224, 10.1039/d3tb00639e. 37306212

[21] Jantrawut P. , Bunrueangtha J. , Suerthong J. , and Kantrong N. , Fabrication and Characterization of Low Methoxyl Pectin/Gelatin/Carboxymethyl Cellulose Absorbent Hydrogel Film for Wound Dressing Applications, Materials. (2019) 12, no. 10, 10.3390/ma12101628, 2-s2.0-85066871103. PMC6567050 31108960

[22] Picone P. , Sabatino M. A. , Ajovalasit A. , Giacomazza D. , Dispenza C. , and Di Carlo M. , Biocompatibility, Hemocompatibility and Antimicrobial Properties of Xyloglucan-Based Hydrogel Film for Wound Healing Application, International Journal of Biological Macromolecules. (2019) 121, 784–795, 10.1016/j.ijbiomac.2018.10.078, 2-s2.0-85055117447. 30342149

[23] Mahmood H. , Khan I. U. , Asif M. et al., *In Vitro* and *In Vivo* Evaluation of Gellan Gum Hydrogel Films: Assessing the Co Impact of Therapeutic Oils and Ofloxacin on Wound Healing, International Journal of Biological Macromolecules. (2021) 166, 483–495, 10.1016/j.ijbiomac.2020.10.206. 33130262

[24] Huanbutta K. and Sangnim T. , Bioadhesive Films for Drug Delivery Systems, Bioadhesives in Drug Delivery, 2020, Wiley, Hoboken, NJ, 99–122.

[25] Suwanpitak K. , Sangnim T. , Sriamornsak P. , Puri V. , Sharma A. , and Huanbutta K. , Development and Validation of a Reliable Reverse-Phase High-Performance Liquid Chromatography Method for Quantifying Triterpenes in *Centella asiatica*: A Step Towards Quality Control of Herbal Products, Science, Engineering and Health Studies. (2025) 19, 10.69598/sehs.19.25050008.

[26] Schmölz L. , Wallert M. , and Lorkowski S. , Optimized Incubation Regime for Nitric Oxide Measurements in Murine Macrophages Using the Griess Assay, Journal of Immunological Methods. (2017) 449, 68–70. 28673787 10.1016/j.jim.2017.06.012

[27] Tan S. C. , Bhattamisra S. K. , Chellappan D. K. , and Candasamy M. , Actions and Therapeutic Potential of Madecassoside and Other Major Constituents of *Centella asiatica*: A Review, Applied Sciences. (2021) 11, no. 18, 10.3390/app11188475.

[28] Wong J. H. , Barron A. M. , and Abdullah J. M. , Mitoprotective Effects of *Centella asiatica* (L.) Urb.: Anti-Inflammatory and Neuroprotective Opportunities in Neurodegenerative Disease, Frontiers in Pharmacology. (2021) 12, 10.3389/fphar.2021.687935. PMC8275827 34267660

[29] Sasmita A. O. , Ling A. P. K. , Voon K. G. L. , Koh R. Y. , and Wong Y. P. , Madecassoside Activates Anti-Neuroinflammatory Mechanisms by Inhibiting Lipopolysaccharide-Induced Microglial Inflammation, International Journal of Molecular Medicine. (2018) 41, no. 5, 3033–3040, 10.3892/ijmm.2018.3479, 2-s2.0-85042767735. 29436598

[30] Ansori A. N. M. , Fadholly A. , Hayaza S. et al., A Review on Medicinal Properties of Mangosteen (*Garcinia mangostana* L.), Research Journal of Pharmacy and Technology. (2020) 13, no. 2, 974–982, 10.5958/0974-360x.2020.00182.1.

[31] Rizaldy D. , Hartati R. , Nadhifa T. , and Fidrianny I. , Chemical Compounds and Pharmacological Activities of Mangosteen (*Garcinia mangostana* L.)—Updated Review, Biointerface Research in Applied Chemistry. (2021) 12, no. 2, 2503–2516.

[32] Salehi B. , Venditti A. , Sharifi-Rad M. et al., The Therapeutic Potential of Apigenin, International Journal of Molecular Sciences. (2019) 20, no. 6, 10.3390/ijms20061305, 2-s2.0-85063006845. PMC6472148 30875872

[33] Aghababaei F. and Hadidi M. , Recent Advances in Potential Health Benefits of Quercetin, Pharmaceuticals. (2023) 16, no. 7, 10.3390/ph16071020. PMC10384403 37513932

[34] Frenț O.-D. , Stefan L. , Morgovan C. M. et al., A Systematic Review: Quercetin—Secondary Metabolite of the Flavonol Class, With Multiple Health Benefits and Low Bioavailability, International Journal of Molecular Sciences. (2024) 25, no. 22, 10.3390/ijms252212091. PMC11594109 39596162

[35] Badhani B. , Sharma N. , and Kakkar R. , Gallic Acid: A Versatile Antioxidant With Promising Therapeutic and Industrial Applications, RSC Advances. (2015) 5, no. 35, 27540–27557, 10.1039/c5ra01911g, 2-s2.0-84925434642.

[36] Sittikijyothin W. , Phonyotin B. , Sangnim T. , and Huanbutta K. , Using Carboxymethyl Gum From *Tamarindus indica* and *Cassia fistula* Seeds With *Chromolaena odorata* Leaf Extract to Develop Antibacterial Gauze Dressing With Hemostatic Activity, Research in Pharmaceutical Sciences. (2021) 16, no. 2, 118–128, 10.4103/1735-5362.310519. 34084199 PMC8102929

[37] Liu J. , Jiang W. , Xu Q. , and Zheng Y. , Progress in Antibacterial Hydrogel Dressing, Gels. (2022) 8, no. 8, 10.3390/gels8080503. PMC9407327 36005104

[38] Mohammad N. A. , Zaidel D. N. A. , Muhamad I. I. , Hamid M. A. , Yaakob H. , and Jusoh Y. M. M. , Optimization of the Antioxidant-Rich Xanthone Extract From Mangosteen (*Garcinia mangostana* L.) Pericarp via Microwave-Assisted Extraction, Heliyon. (2019) 5, no. 10, 10.1016/j.heliyon.2019.e02571, 2-s2.0-85073025285. PMC6812211 31667409

[39] Ukaegbu K. , Allen E. , and Svoboda K. K. , Reactive Oxygen Species and Antioxidants in Wound Healing: Mechanisms and Therapeutic Potential, International Wound Journal. (2025) 22, no. 5, 10.1111/iwj.70330. PMC12034374 40288766

[40] Gunter N. V. , Teh S. S. , Lim Y. M. , and Mah S. H. , Natural Xanthones and Skin Inflammatory Diseases: Multitargeting Mechanisms of Action and Potential Application, Frontiers in Pharmacology. (2020) 11, 10.3389/fphar.2020.594202. PMC7793909 33424605

[41] Mohan S. , Syam S. , Abdelwahab S. I. , and Thangavel N. , An Anti-Inflammatory Molecular Mechanism of Action of α-Mangostin, the Major Xanthone From the Pericarp of *Garcinia mangostana*: An *In Silico, In Vitro* and *In Vivo* Approach, Food & Function. (2018) 9, no. 7, 3860–3871, 10.1039/c8fo00439k, 2-s2.0-85050541065. 29953154

[42] Ali-Seyed M. and Vijayaraghavan K. , Nutraceuticals for Wound Healing: A Special Focus on *Chromolaena odorata* as Guardian of Health With Broad Spectrum of Biological Activities, Nutraceuticals in Veterinary Medicine, 2019, Springer, Berlin, 541–562.

[43] Khairunnisa N. Z. , Massi M. N. , Sunarno I. , Hami F. , Usman A. N. , and Prihantono P. , The Potential of Processing *Chromolaena odorata* Leaves in Solving Health Issues: A Review, BIO Web of Conferences. (2024) 96, 10.1051/bioconf/20249601015.

[44] Sun B. , Wu L. , Wu Y. et al., Therapeutic Potential of *Centella asiatica* and Its Triterpenes: A Review, Frontiers in Pharmacology. (2020) 11, 10.3389/fphar.2020.568032. PMC7498642 33013406

[45] Feng Y. , Guo W. , Hu L. , Yi X. , and Tang F. , Application of Hydrogels as Sustained-Release Drug Carriers in Bone Defect Repair, Polymers. (2022) 14, no. 22, 10.3390/polym14224906. PMC9695274 36433033

[46] Witkowska K. , Paczkowska-Walendowska M. , Garbiec E. , and Cielecka-Piontek J. , Topical Application of *Centella asiatica* in Wound Healing: Recent Insights Into Mechanisms and Clinical Efficacy, Pharmaceutics. (2024) 16, no. 10, 10.3390/pharmaceutics16101252. PMC11510310 39458583

[47] Seshadri V. D. , Vijayaraghavan P. , Kim Y.-O. et al., *In Vitro* Antioxidant and Cytotoxic Activities of Polyherbal Extracts From *Vetiveria zizanioides*, *Trichosanthes cucumerina*, and *Mollugo cerviana* on Hela and MCF-7 Cell Lines, Saudi Journal of Biological Sciences. (2020) 27, no. 6, 1475–1481, 10.1016/j.sjbs.2020.04.005. 32489283 PMC7254031

[48] Lei L. , Bai Y. , Qin X. , Liu J. , Huang W. , and Lv Q. , Current Understanding of Hydrogel for Drug Release and Tissue Engineering, Gels. (2022) 8, no. 5, 10.3390/gels8050301. PMC9141029 35621599

[49] Namuga C. , Ocan M. , Kinengyere A. A. et al., Efficacy of Nano Encapsulated Herbal Extracts in the Treatment of Induced Wounds in Animal Models: A Systematic Review Protocol, Systematic Reviews. (2023) 12, no. 1, 10.1186/s13643-023-02370-7. PMC10652619 37968731

[50] Aziz N. A. , Mohamad M. , Mohsin H. F. , Mohamad Nor Hazalin N. A. , and Abdul Hamid K. , The Pharmacological Properties and Medicinal Potential of *Chromolaena odorata*: A Review, International Journal of Pharmaceuticals, Nutraceuticals and Cosmetic Science. (2020) 2, 30–41, 10.24191/ijpnacs.v2.04.

[51] Nakamura J. , Hikichi T. , Watanabe K. et al., Efficacy of Sodium Carboxymethylcellulose Compared to Sodium Hyaluronate as Submucosal Injectant for Gastric Endoscopic Submucosal Dissection: A Randomized Controlled Trial, Digestion. (2021) 102, no. 5, 753–759, 10.1159/000513148. 33611330

[52] Basiak E. , Lenart A. , and Debeaufort F. , How Glycerol and Water Contents Affect the Structural and Functional Properties of Starch-Based Edible Films, Polymers. (2018) 10, no. 4, 10.3390/polym10040412, 2-s2.0-85045086014. PMC6415220 30966447

[53] Arefian M. , Hojjati M. , Tajzad I. , Mokhtarzade A. , Mazhar M. , and Jamavari A. , A Review of Polyvinyl Alcohol/Carboxymethyl Cellulose (PVA/CMC) Composites for Various Applications, Journal of Composites and Compounds. (2020) 2, no. 3, 69–76.

[54] Chen C.-h. , Torrents A. , Kulinsky L. et al., Mechanical Characterizations of Cast Poly(3,4-ethylenedioxythiophene): Poly(Styrenesulfonate)/Polyvinyl Alcohol Thin Films, Synthetic Metals. (2011) 161, no. 21-22, 2259–2267, 10.1016/j.synthmet.2011.08.031, 2-s2.0-80055080501.

[55] Ding C. , Ma W. , and Zhong J. , The Influence of Microcrystalline Structure and Crystalline Size on Visible Light Transmission of Polyvinyl Alcohol Optical Films, Optical Materials. (2024) 147, 10.1016/j.optmat.2023.114627.

[56] Chau A. L. , Getty P. T. , Rhode A. R. , Bates C. M. , Hawker C. J. , and Pitenis A. A. , Superlubricity of pH-Responsive Hydrogels in Extreme Environments, Frontiers in Chemistry. (2022) 10, 10.3389/fchem.2022.891519. PMC9405656 36034669

[57] Gefen A. , Not all Superabsorbent Wound Dressings Are Born Equal: Theory and Experiments, Journal of Wound Care. (2021) 30, no. 9, 738–750, 10.12968/jowc.2021.30.9.738. 34554841

[58] Hoti G. , Caldera F. , Cecone C. et al., Effect of the Cross-Linking Density on the Swelling and Rheological Behavior of Ester-Bridged β-Cyclodextrin Nanosponges, Materials. (2021) 14, no. 3, 10.3390/ma14030478. PMC7864023 33498322

[59] Asy-Syifa N. , Waresindo W. X. , Edikresnha D. , Suciati T. , and Khairurrijal K. , The Study of the Swelling Degree of the PVA Hydrogel With Varying Concentrations of PVA, Journal of Physics: Conference Series. (2022) 2243, no. 1, 10.1088/1742-6596/2243/1/012053.

[60] Bidgoli H. , Zamani A. , and Taherzadeh M. J. , Effect of Carboxymethylation Conditions on the Water-Binding Capacity of Chitosan-Based Superabsorbents, Carbohydrate Research. (2010) 345, no. 18, 2683–2689, 10.1016/j.carres.2010.09.024, 2-s2.0-78649334962. 20971451

[61] Bialik-Wąs K. , Pluta K. , Malina D. , Barczewski M. , Malarz K. , and Mrozek-Wilczkiewicz A. , The Effect of Glycerin Content in Sodium Alginate/Poly(Vinyl Alcohol)-Based Hydrogels for Wound Dressing Application, International Journal of Molecular Sciences. (2021) 22, no. 21, 10.3390/ijms222112022. PMC8584732 34769449

